# Supplementary material for: ACKR2 in hematopoietic precursors as a checkpoint of neutrophil release and anti-metastatic activity
Source: Nat Commun. 2018 Feb 14;9:676. doi: 10.1038/s41467-018-03080-8 (PMC5813042; doi:10.1038/s41467-018-03080-8)
Supplement: Supplementary file 1 — Supplementary Information [file 41467_2018_3080_MOESM1_ESM.pdf]

Supplementary material for:

ACKR2 IN HEMATOPOIETIC PRECURSORS AS A CHECKPOINT OF NEUTROPHIL RELEASE AND ANTIMETASTATIC ACTIVITY

Matteo Massara[1,2,†], Ornella Bonavita[1,2,†], Benedetta Savino[1,2,†], Nicoletta Caronni[1,2,†], Valeria Mollica Poeta[1,3], Marina Sironi[1], Elisa Setten[1,2], Camilla Recordati[4], Laura Crisafulli[1,5], Francesca Ficara[1,5], Alberto Mantovani[1,3,6], Massimo Locati[1,2], Raffaella Bonecchi[1,3*]

[1]Humanitas Clinical and Research Center, via Manzoni 56, 20089, Rozzano (MI), Italy; [2]Department of Medical Biotechnologies and Translational Medicine, Università degli Studi di Milano, Via Fratelli Cervi, 93, 20090 Segrate (MI), Italy; [3]Department of Biomedical Sciences, Humanitas University, Via Rita Levi Montalcini, 20090, Pieve Emanuele (MI), Italy; [4]Fondazione Filarete, viale Ortles 22/4, 20139, Milano, Italy; [5]Milan Unit, Istituto di Ricerca Genetica e Biomedica, CNR, via Manzoni 56, 20089 Rozzano (MI); [6] The William Harvey Research Institute, Queen Mary University of London, Charterhouse Square, London EC1M 6BQ, UK.

[†] = these authors equally contributed

*** Corresponding author:**
Raffaella Bonecchi
Humanitas Clinical and Research Center
Humanitas University
Via Manzoni 113
20089 Rozzano, Italy
Tel +39.02.8224.5117
E-mail: raffaella.bonecchi@humanitasresearch.it

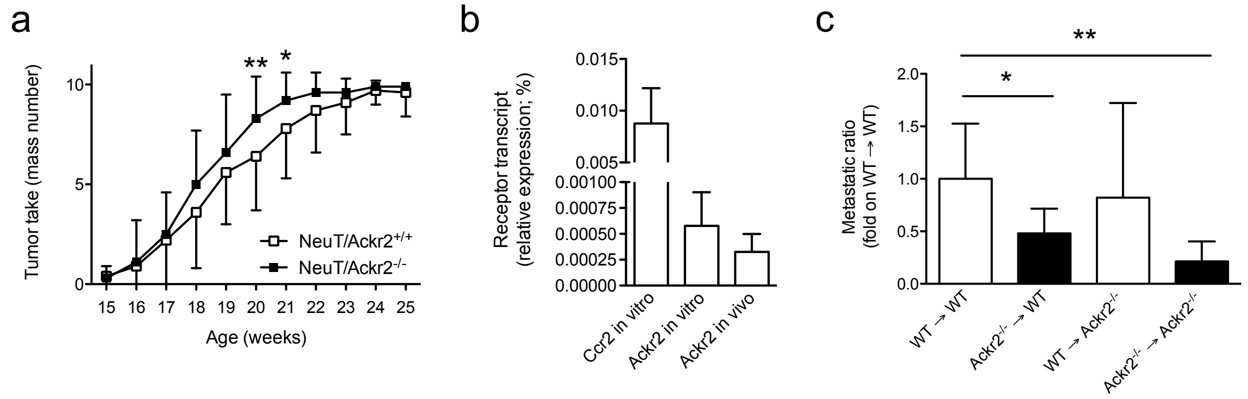

Supplementary Figure 1: Metastasis protection is given by lack of ACKR2 by hematopoietic cells.

a) *NeuT/Ackr2*$^{+/+}$ (white squares) and *NeuT/Ackr2*$^{-/-}$ (black squares) mice were evaluated for tumor take calculated as described in the Materials and Methods section (n = 42 for *NeuT/Ackr2*$^{+/+}$ and 23 for *NeuT/Ackr2*$^{-/-}$ mice, respectively) b) qPCR analysis of *Ackr2* and *Ccr2* expression by 4T1 cells in vitro and sorted from a tumor mass after 21 days from injection as CD45^{-}/CD31^{-}/Podoplanin^{-}. Data are relative to *β-actin* expression (n = 4 for in vitro cancer cell expression and n = 3 WT mice for in vivo cancer cell expression). c) Metastatic ratio of WT and *Ackr2*$^{-/-}$ mice reconstituted with either WT (white columns) or *Ackr2*$^{-/-}$ BM (black columns) after 28 days from orthotopic 4T1 injection calculated as described in the Materials and Methods section (n = 6 for WT and 4 for *Ackr2*$^{-/-}$ recipient mice, respectively). Data are represented as mean (SD). p value was generated using the unpaired t test. * = $p < 0.05$, ** = $p < 0.01$, *** = $p < 0.001$.

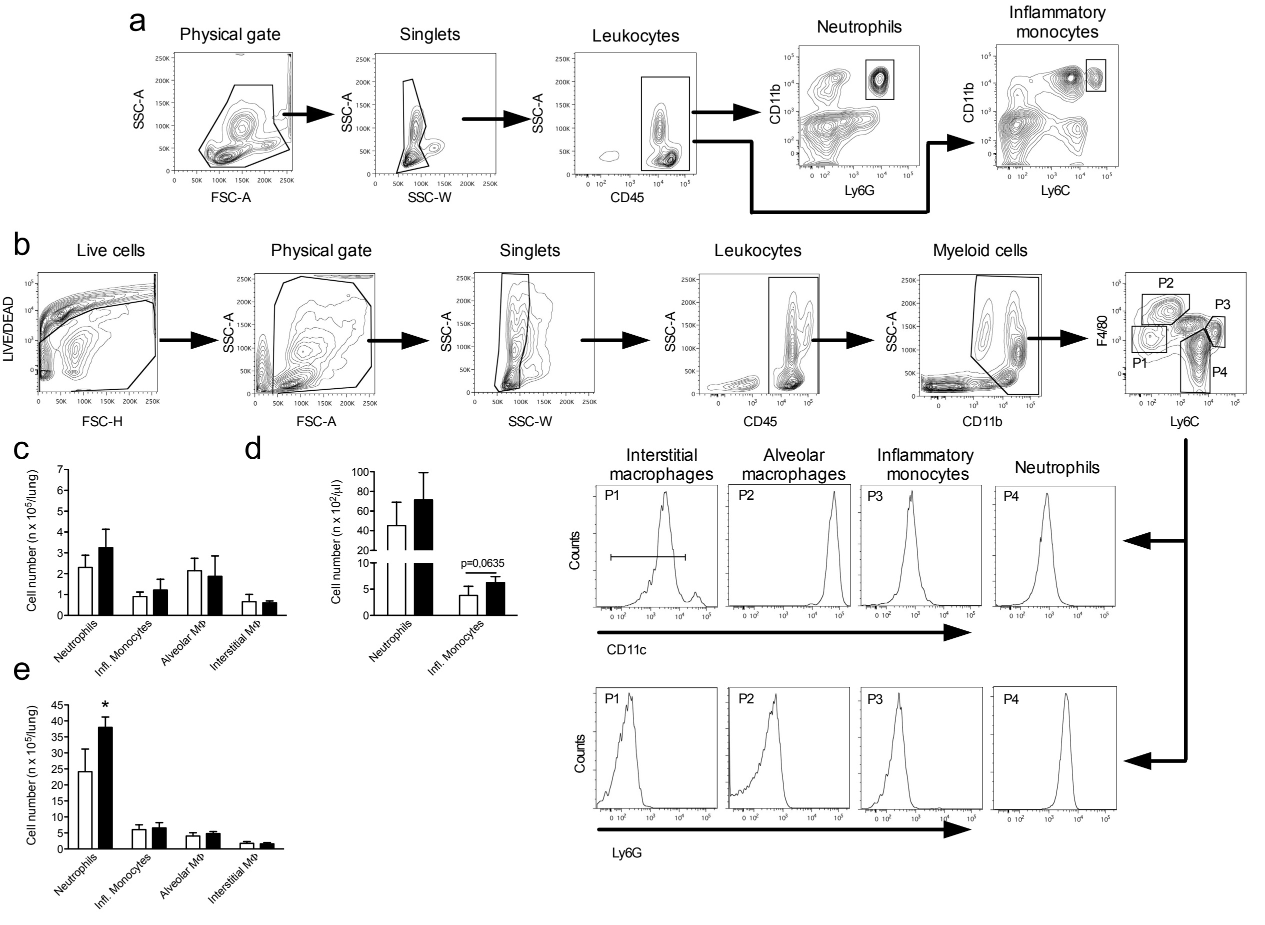

Supplementary Figure 2: Gating strategy for the identification of blood and lung neutrophils and monocytes

a) Gating strategy to identify circulating neutrophils (CD45$^+$/CD11b$^+$/Ly6G$^+$) and inflammatory monocytes (CD45$^+$/CD11b$^+$/Ly6C^{hi}). b) Gating strategy for the identification of interstitial macrophages (P1; CD11b$^+$/F4/80^{int}/Ly6C$^-$/CD11c$^-$/Ly6G$^-$) alveolar macrophages (P2: CD11b^{low}/F4/80^{hi}/Ly6C^{int}/CD11c$^+$/Ly6G$^-$), inflammatory monocytes (P3; CD11b$^+$/F4/80^{int}/Ly6C^{hi}/CD11c$^-$/Ly6G$^-$), and neutrophils (P4; CD11b$^+$/F4/80$^-$/Ly6C^{int}/CD11c$^-$/Ly6G$^+$) in the lungs of tumor-bearing mice. c) Absolute number of neutrophils, inflammatory monocytes, alveolar and infiltrating macrophages in WT (white columns) and *Ackr2*$^{-/-}$ (black columns) lungs taken from unchallenged mice (n = 4 for WT and 6 for *Ackr2*$^{-/-}$ mice, respectively). d) Absolute number of circulating neutrophils and inflammatory monocytes in WT (white columns) and *Ackr2*$^{-/-}$ (black columns) mice and e) absolute number of neutrophils, inflammatory monocytes, alveolar and infiltrating macrophages in WT (white columns) and *Ackr2*$^{-/-}$ (black columns) lungs 14 days after orthotopic injection of 4T1 cells (n = 4 for WT and 6 for *Ackr2*$^{-/-}$ mice, respectively). Data are represented as mean (SD). p value was generated using the unpaired t test. * = $p < 0.05$, ** = $p < 0.01$, *** = $p < 0.001$.

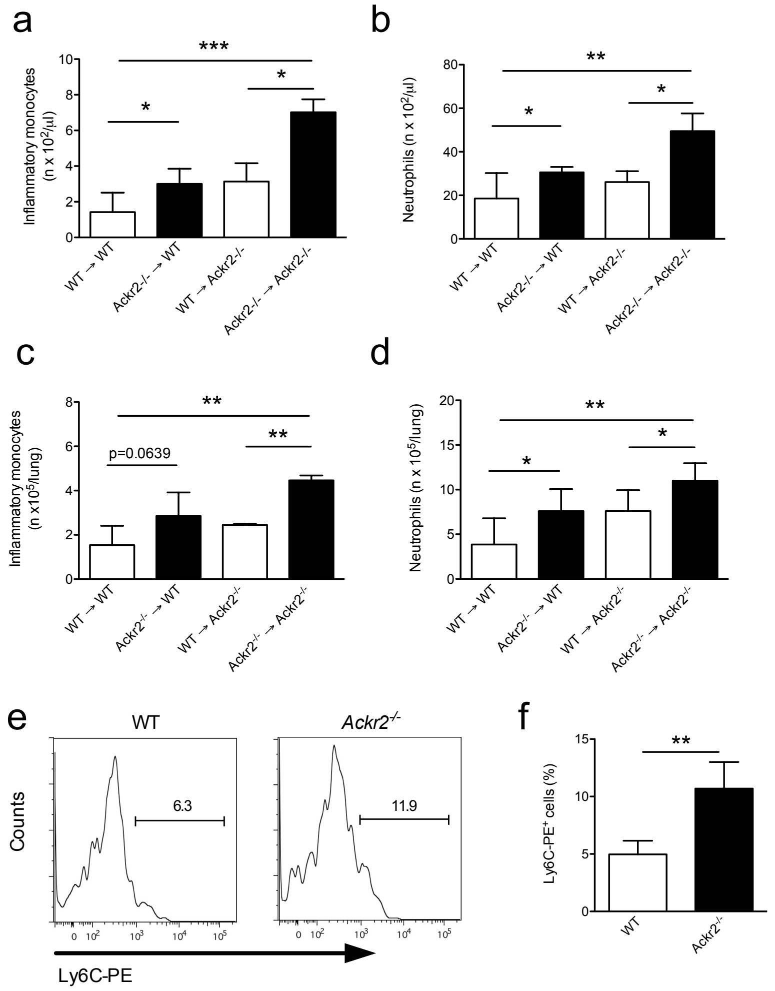

Supplementary Figure 3: Hematopoietic expression of ACKR2 results in increased number of monocytes and neutrophils in blood and lungs

Absolute number of circulating inflammatory monocytes (a) and neutrophils (b) in the blood of Balb/c WT and *Ackr2*$^{-/-}$ mice reconstituted with either WT (white columns) or *Ackr2*$^{-/-}$ BM (black columns) after i.p. injection of CCL3L1 (n = 6 for WT and 4 for *Ackr2*$^{-/-}$ recipient mice, respectively). Absolute number of lung infiltrating inflammatory monocytes (c) and neutrophils (d) in WT and *Ackr2*$^{-/-}$ mice reconstituted with either WT (white columns) or *Ackr2*$^{-/-}$ BM (black columns) after i.p. injection of CCL3L1 (n = 6 for WT and 4 for *Ackr2*$^{-/-}$ recipient mice, respectively). e) Representative histograms of FACS analysis of BM WT and *Ackr2*$^{-/-}$ Ly6C-PE positive monocytes 1 h after i.p. injection of CCL3L1. Ly6C-PE antibody was injected i.v. 2 min before the end of the experiment. Negative gate was set on FMO control. f) Percentage of Ly6C-PE labelled cells (calculated on FMO samples) on total BM monocytes ($CD45^+/CD11b^+/Gr1^+/Ly6G^-$) (n = 4 for both WT and *Ackr2*$^{-/-}$ mice). Data are represented as mean (SD). p value was generated using the unpaired t test. * = $p < 0.05$, ** = $p < 0.01$, *** = $p < 0.001$.

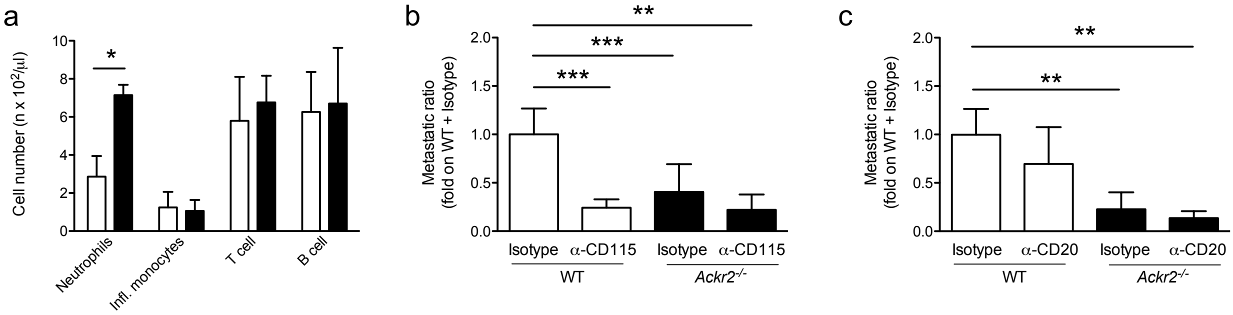

Supplementary Figure 4: Monocyte and B cell depletions did not revert metastasis protection in *Ackr2*$^{-/-}$ mice

a) Absolute number of circulating neutrophils, inflammatory monocytes, T cells and B cells in the blood of WT and *Ackr2*$^{-/-}$ mice 10 days after i.v. injection with B16F10 cells (n = 5 for both WT and *Ackr2*$^{-/-}$ mice). b – c) Metastatic ratio in WT and Ackr2$^{-/-}$ mice depleted for monocytes with α-CD115 (b) (n = 5 for WT/Isotype, Ackr2$^{-/-}$ /Isotype and Ackr2$^{-/-}$/α-CD115 and 6 for WT/α-CD115) and B cells with α-CD20 (c) (n = 4 for WT/Isotype, 6 for WT/α-CD20, 3 for Ackr2$^{-/-}$/Isotype, 3 for Ackr2$^{-/-}$/α-CD20) 10 days after i.v. injection of B16F10 cells. Data are represented as mean (SD). p value was generated using the unpaired t test. * = $p < 0.05$, ** = $p < 0.01$, *** = $p < 0.001$.

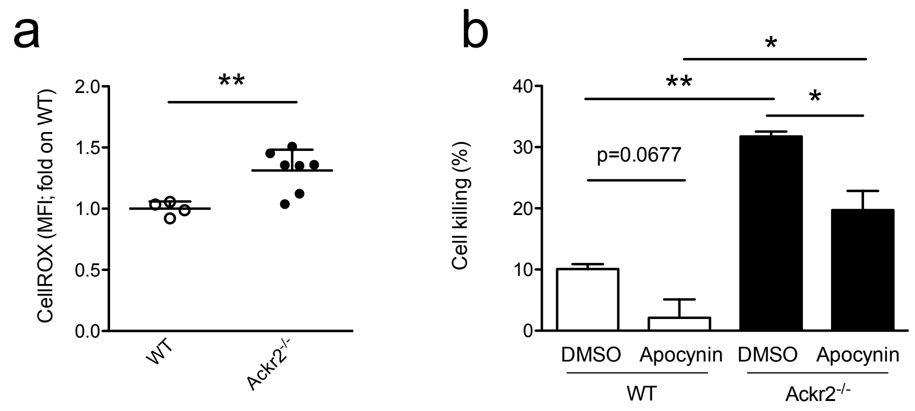

Supplementary Figure 5: *Ackr2$^{-/-}$* neutrophils show increased cell killing activity

a) MFI of CellROX emission by WT and *Ackr2$^{-/-}$* neutrophils. Data are normalized on MFI of WT neutrophils (n = 4 for WT and 7 for *Ackr2$^{-/-}$*). b) 4T1-luc cells killing by magnetically sorted BM neutrophils taken from unchallenged WT (white columns) and *Ackr2$^{-/-}$* (black columns) mice. Where indicated Apocynin (100 μM) was added (n = 3 for both WT and *Ackr2$^{-/-}$*), two independent experiments). Data are represented as mean (SD). p value was generated using the unpaired t test. * = $p < 0.05$, ** = $p < 0.01$, *** = $p < 0.001$.

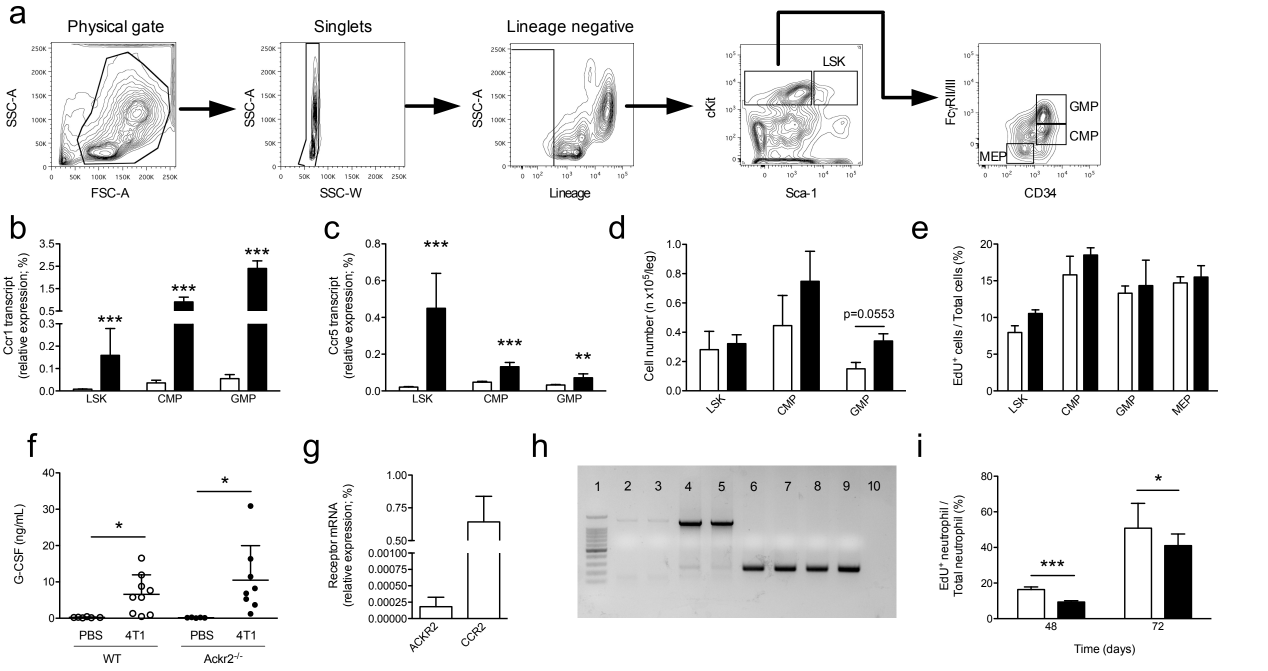

Supplementary Figure 6: HPCs in the BM of WT and *Ackr2*$^{-/-}$ mice and Ackr2 expression in mock and Ackr2 transfected HL-60 cells

a) Gating strategy for the identification of LSK, GMP, CMP and MEP in the BM. qPCR analysis of the expression of *Ccr1* (b) and *Ccr5* (c) on sorted HPCs taken from the BM of WT (white columns) and *Ackr2*$^{-/-}$ (black columns) mice. All qPCR data are relative to β-actin expression (n = 7 for both WT and *Ackr2*$^{-/-}$ mice). D) Absolute number of HPCs in the BM of WT and *Ackr2*$^{-/-}$ mice calculated by FACS analysis (n = 6 for both WT and *Ackr2*$^{-/-}$ mice). e) Percentage of proliferating hematopoietic progenitors measured by FACS as EdU^{+} cells. Gate was set on negative control (n = 3 for both WT and *Ackr2*$^{-/-}$ mice). f) Serum levels of G-CSF in WT and *Ackr2*$^{-/-}$ mice taken from mice 21 days after orthotopic injection with 4T1 cells or PBS (n = 6 for WT/PBS, 9 for WT/4T1, 5 for *Ackr2*$^{-/-}$/PBS, 8 for *Ackr2*$^{-/-}$/4T1). g) qPCR analysis of the expression of ACKR2 and CCR2 in HL-60 cells. Data are normalized on *β-actin* expression. h) RT-PCR analysis of *ACKR2* (lanes 2-5) and *GAPDH* (lanes 6-9) expression on mock (lanes 2,3,6,7) and ACKR2 transfected (lanes 4,5,8,9) HL-60 cells. Lane 10 shows the negative control. i) Percentage of EdU^{+} neutrophils on total neutrophils measured by FACS analysis after 48 and 78 hours from EdU injection. Gate was set on negative control (n = 6 for WT mice, 5 for *Ackr2*$^{-/-}$ mice). Data are represented as mean (SD). p value was generated using the unpaired t test. * = $p < 0.05$, ** = $p < 0.01$, *** = $p < 0.001$.

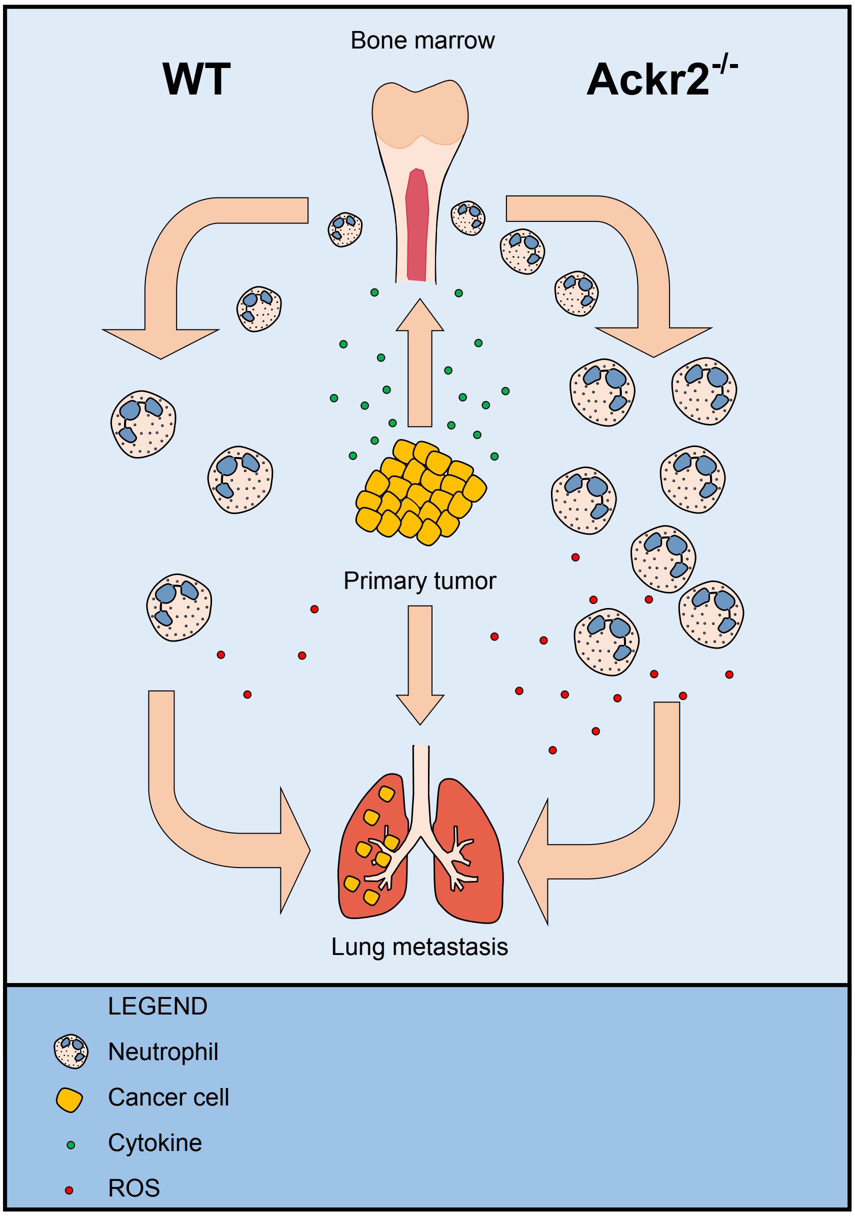

Supplementary Figure 7: ACKR2 inhibits the release of anti-metastatic neutrophils. ACKR2 activity in HPCs inhibits the expression of CC chemokine receptors, thus limiting the mobilization and maturation of neutrophils. Neutrophils released from the BM in *Ackr2*$^{-/-}$ mice have increased tumor killing ability.

Supplementary Table 1: List of antibodies.

Antigen	Fluorochrome	Clone	Supplier	Catalog #	Working dilution
CD45	PerCP	30-F11	Biolegend	103130	1:50
CD45	V450	30-F11	BD Bioscience	560501	1:50
CD45	BV605	30-11	BD Bioscience	563053	1:50
CD11b	Pacific Blue	M1/70	Biolegend	101224	1:50
CD11b	PE	M1/70	BD Bioscience	557397	1:50
CD11b	PerCP-Cy5.5	M1/70	BD Bioscience	550993	1:50
CD11c	APC	N418	eBioscience	17-0114-82	1:100
CD31	BV421	390	Biolegend	102424	1:100
Podoplanin	PeCy7	8.1.1	Biolegend	127412	1:100
Ly6G	PeCy7	1A8	BD Bioscience	560601	1:100
Ly6G	FITC	1A8	BD Bioscience	551460	1:100
Ly6C	FITC	AL-21	BD Bioscience	553104	1:100
Ly6C	PE	AL-21	BD Bioscience	560592	1:59
F4/80	PE	CI:A3-1	AbD Serotec	MCA497PE	1:50
Gr-1	APC	RB6-8C5	BD Bioscience	553129	1:50
CCR2		Purified	Gift Matthias Mack		1:370
anti-rat IgG2b	Biotin	G15-337	BD Bioscience	553883	1:50
Streptavidin	PE		BD Bioscience	554061	1:50
Streptavidin	eF450		eBioscience	48-4317-82	1:50
CXCR4	PE	2B11	eBioscience	12-9991-82	1:25
Sca-1	PeCy7	D7	eBioscience	25-5981-82	1:100
c-Kit	APC-eFluor780	2B8	eBioscience	47-1172-82	1:100
CD34	FITC	RAM34	BD Bioscience	553733	1:100
FcγRII/III	PerCP-Cy5.5	93	eBioscience	45-0161-82	1:100
Lineage cocktail	eFluor 450	17A2, RA3-6B2, M1/70, TER-119, RB6-8C5	eBioscience	88-7772-72	1:5

Supplementary Table 2: List of Taqman probes for qPCR.

Gene name	Host	Code
Ackr2	Murine	Mm_00445551_m1
Ccr1	Murine	Mm01216147_m1
Ccr2	Murine	Mm_00438270_m1
Ccr5	Murine	Mm04207879_m1
Cxcr4	Murine	Mm_01292123_m1
Vegfa	Murine	Mm00437306_m1
Tnf-α	Murine	Mm00443258_m1
Alox5	Murine	Mm01182747_m1
Arg1	Murine	Mm_00475988_m1
Gapdh	Murine	Mm99999915_g1
β-actin	Murine	Mm_00607939_s1
ACKR2	Human	Hs_00174299_m1
CCR2	Human	Hs00704702_s1
CXCR4	Human	Hs_00607978_s1
CD11b	Human	Hs_00355885_m1
GAPDH	Human	Hs_99999905_m1

Supplementary Table 3: Metastasis score of *NeuT/Ackr2*$^{+/+}$, *NeuT/Ackr2*$^{-/-}$ mice and Balb/c mice orthotopically injected with 4T1 cells and treated as indicated. The metastasis score was calculated as described in the Material and Methods section (MR = metastatic ratio = *Ackr2*$^{-/-}$ metastasis score / WT metastasis score).

		WT			*Ackr2*$^{-/-}$			
MODEL	FIGURE	Mean	SD	n	Mean	SD	n	MR
NeuT	1C	33.1	1.0	26	17	5.6	16	0.51
4T1	1F	13.5	1.0	14	5.3	2.3	13	0.17
4T1 66cl4	1F	7	1.8	4	8.8	5.6	4	1.26
4T1 IgG	4C	14.0	4.0	3	3.3	1.5	5	0.24
4T1 α-Ly6G	4C	2.3	0.6	3	7.7	3.5	3	3.35

Supplementary Table 4: Number of metastasis in WT and *Ackr2*$^{-/-}$ mice i.v. injected with B16F10 cells and treated as indicated (MR = metastatic ratio = *Ackr2*$^{-/-}$ metastasis number / WT metastasis number).

		WT			*Ackr2*$^{-/-}$			
Treatment	FIGURE	Mean	SD	n	Mean	SD	n	MR
-	4A	235.1	62.3	14	102.8	54.3	8	0.41
IgG	4B	92.0	38.4	6	28.7	5.0	3	0.31
α-Ly6G	4B	28.0	17.4	5	74.5	44.7	4	2.66
IgG	S4A	157.6	42.3	5	72.8	44.3	6	0.46
α-CD115	S4A	38.2	13.8	5	34.8	24.8	5	0.91
IgG	S4B	75.0	20.0	4	17.0	13.0	3	0.23
α-CD20	S4B	52.2	28.3	6	10.3	5.5	3	0.20

Supplementary Table 5: Number of metastasis in WT mice i.v. injected with B16F10 cells and adoptively transferred with neutrophils of the indicated genotype.

	PBS			WT neutrophils			*Ackr2*$^{-/-}$ neutrophils		
FIGURE	Mean	SD	n	Mean	SD	n	Mean	SD	N
4D	230.8	36.3	5	245.4	31.7	9	94.3	57.4	8
